# Ten-Year Monitored Natural Recovery of Lead-Contaminated Mine Tailing in Klity Creek, Kanchanaburi Province, Thailand

**DOI:** 10.1289/EHP215

**Published:** 2016-05-08

**Authors:** Tanapon Phenrat, Ashijya Otwong, Aphichart Chantharit, Gregory V. Lowry

**Affiliations:** 1 Research Unit for Integrated Natural Resources Remediation and Reclamation (IN3R), Department of Civil Engineering, and; 2 Center of Excellence for Sustainability of Health, Environment and Industry (SHEI), Faculty of Engineering, Naresuan University, Phitsanulok, Thailand; 3 ENLAWTHAI Foundation, Bangkok, Thailand; 4 Faculty of Law, Naresuan University, Phitsanulok, Thailand; 5 Center for Environmental Implications of Nanotechnology (CEINT), and; 6 Department of Civil and Environmental Engineering, Carnegie Mellon University, Pittsburgh, Pennsylvania, USA

## Abstract

**Background:**

Klity Creek has become Thailand’s first official remediation ordered by the court in 2013, 15 years after the spill of lead (Pb)-contaminated mine tailing into the creek. The Pollution Control Department (PCD) decided to restore the creek through monitored natural recovery (MNR) since 2006 but has not been successful. Interestingly, the most recent remediation plan in 2015 will still apply MNR to five out of the seven portions of the creek, despite no scientific feasibility evaluation of using MNR to restore the creek.

**Objective:**

This study qualitatively and quantitatively evaluated the feasibility of using MNR to clean up the creek in order to protect the Klity children from excess Pb exposure.

**Methods:**

We analyzed the physical and chemical transformation of Pb contaminated sediment in the creek and developed a remedial action goal and cleanup level using the Integrated Exposure Uptake Biokinetic model (IEUBK). We empirically determined the natural recovery (NR) potentials and rates using 10 years of data monitoring the water and sediment samples from eight monitoring stations (KC1 to KC8).

**Results:**

Klity Creek has NR potential for water except at KC2, which is closest to the spill and the other improperly managed Pb sources. However, the creek has no NR potential for sediment except at the KC8 location (NR rate = 11.1 ± 3.0 × 10–3 month–1) farthest from the spill.

**Conclusion:**

The MNR method is not suitable to use as the sole remedial approach for Klity Creek (KC2 to KC7). Although MNR is applicable at KC8, it may require up to 377 ± 76 years to restore the sediment to the background Pb concentration.

**Citation:**

Phenrat T, Otwong A, Chantharit A, Lowry GV. 2016. Ten-year monitored natural recovery of lead-contaminated mine tailing in Klity Creek, Kanchanaburi Province, Thailand. Environ Health Perspect 124:1511–1520; http://dx.doi.org/10.1289/EHP215

## Introduction

**Figure 1:**
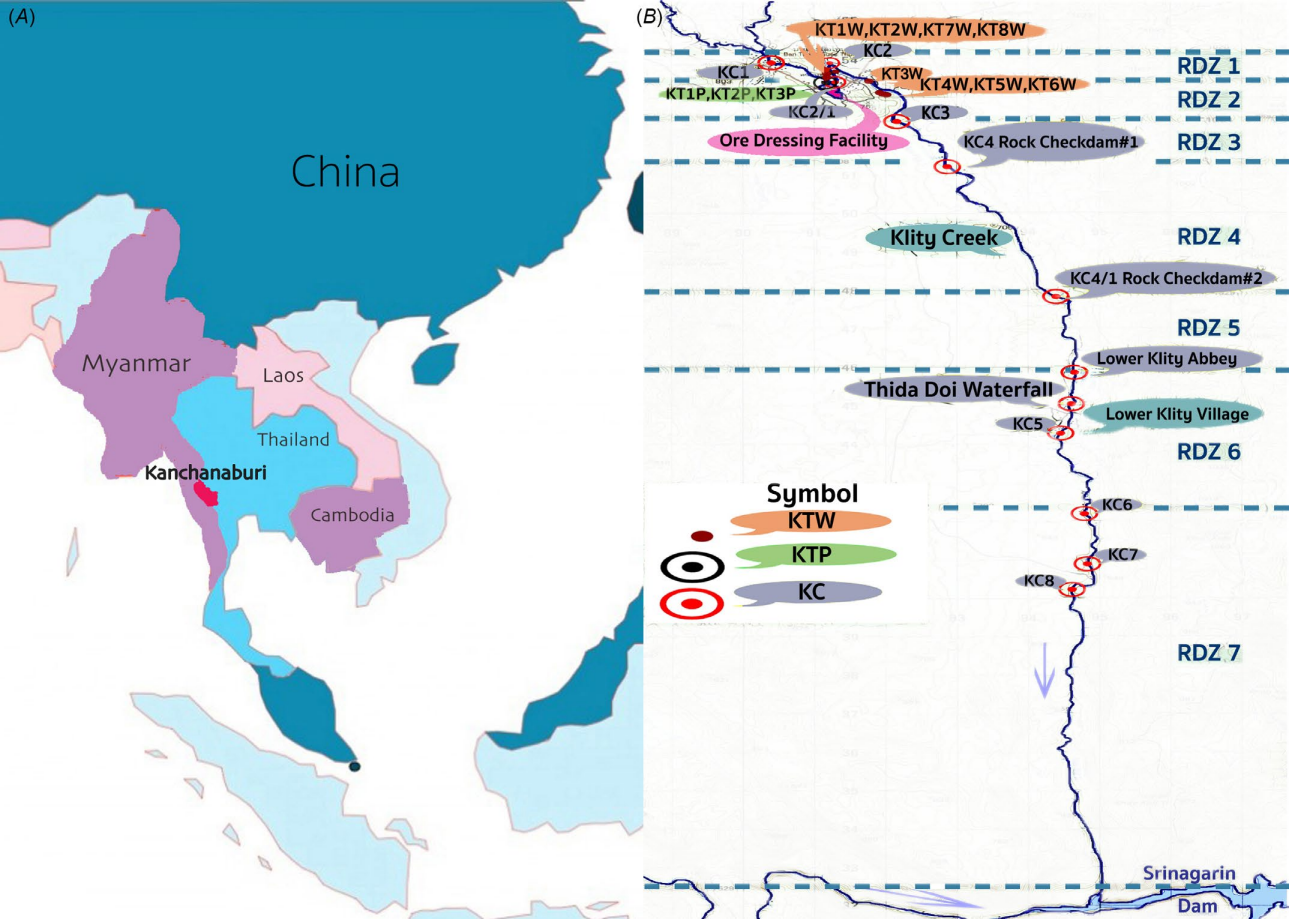
(*A*) A map of Kanchanaburi province, Thailand (red) and (*B*) a map of Klity Creek with the location of monitoring stations (KC1 to KC8), abandoned LCMT ponds (KT1P to KT3P), and surface impoundments, which contain dredged LCMT (KT1W to KT8W) (image courtesy of Raviwan Rakthinkamnerd).

Lead (Pb) contamination in Klity Creek in Kanchanaburi’s Thong Pha Phum district (Figure 1) is perhaps the most infamous contaminated mining site in Thailand. It has continuously gained media and public attention for more than 17 years (Pearshouse 2014), since the spill of the Pb-contaminated mine tailing (LCMT) (Panichayapichet et al. 2008; Pedall et al. 1999), which first came under media spotlight in 1998. Klity Creek is 28 km long and is the only water source for more than 400 villagers. A Pb flotation plant established in 1967 was upstream from the creek. In 1998, the facility reported that torrential rain damaged an old retaining pond causing the spill of around 17,540 metric tons of LCMT into the Klity Creek watershed (Panichayapichet et al. 2008; Pedall et al. 1999). According to the survey in 1999, Pb concentrations in the Klity Creek’s sediments and riverbank were as high as 113,586 mg/kg and 93,388 mg/kg, respectively, at KC2 (Figure 1), which is closest to the spill incident (Pedall et al. 1999).

In addition, 3 mine tailing ponds (KT1P, KT2P, and KT3P) in the vicinity of the ore dressing facility are upstream of Klity Creek and contain around 889,108 metric tons of LCMT at the maximum concentration of 214,000 mg/kg (PCD 2014). Furthermore, more than 14 improper surface impoundments (for example, KTW1 to KTW8 in Figure 1), burying as much as 58,940 tons of LCMT at the maximum Pb concentration of 181,960 mg/kg are in residential areas along the creek (PCD 2014). Having neither an appropriate impermeable liner nor monitoring wells, these ponds and surface impoundments may serve as continuous sources releasing Pb to the creek over many years. Lead is a non-essential, poisonous metal posing both carcinogenic and non-carcinogenic health risks. Since the nervous system is the most sensitive target of Pb exposure, chronic exposure of Pb at low levels could result in a spectrum of nerve injury, renal impairment, immunotoxicity, and toxicity to the reproductive organs (Bellinger and Bellinger 2006; WHO 2010). Children have a greater risk of exposure and greater susceptibility to Pb poisoning than adults do, presumably due to their vulnerable lifestyle and biological susceptibility. Unsurprisingly, a study reported that Klity villagers especially children tended to score lower on IQ tests and more frequently reported illnesses, such as nausea, vomiting, abdominal pain, constipation, concentration problems, muscle pains, headaches, insomnia, and memory loss (Pusapukdepob et al. 2007), which are the symptoms associated with Pb exposure, than the villagers in nearby communities. For this reason, the Ministry of Public Health has tried to reduce risk of the Klity villagers by prohibiting the consumption of water and fish from the creek since 2002. Nevertheless, it might not be effective. According to recent community-based research (Pengkham 2015), Klity villagers still eat fish and drink water from the creek, as they have no other choices. Thus, restoration of Klity Creek is an essential step to protect Klity villagers from further Pb exposure.

The Pollution Control Department (PCD) has been in charge of supervising, monitoring, and conducting remedial actions since the discovery of the incident (Pearshouse 2014; Pusapukdepob et al. 2007). The PCD ordered the company to remediate the creek by dredging lead-contaminated sediment (LCS) and constructing two rock-check dams (KC4 and KC4/1, Figure 1) to block LCS from migration to villagers downstream. However, the PCD could not enforce the company to follow the remediation plan. Instead, the company stopped cleaning up the creek by dredging. This was the main reason that 22 Klity villagers entered the lawsuit in the Central Administrative Court, accusing the PCD of negligence in 2004 (S. Trongngam, oral communication, March 2015).

Eight months later, the PCD decided to stop the cleanup of the creek by dredging because of the belief that dredging would further spread LCS and increase Pb exposure to Klity villagers. Instead, PCD proposed using monitored natural recovery (MNR) to restore the creek (PCD 2008). Nevertheless, to Klity villagers and non-governmental organizations, MNR means that after a 5-year fruitless remediation, PCD would do no action and let nature resolve itself. The lawyer in this case raised an objection to the court that PCD decided to change their plan without sufficient supporting research and public participation (S. Trongngam, oral communication, March 2015).

The MNR method relies on natural processes to reduce unacceptable risk from contamination. Important processes include burial and in-place dilution following deposition of clean sediment and biodegradation or abiotic transformation processes, which convert the contaminants to less toxic forms (Fuchsman et al. 2014). The MNR method was effectively used in remediation trains to restore sediment contaminated with organic pollutants (Fuchsman et al. 2014). It was also used to restore sediment contaminated with metals, but this is much more rare (Moore and Langner 2012). However, not all sediment contamination cases are applicable for MNR. To use MNR, a remediation engineer must demonstrate that natural processes in the site can achieve containment and reduction of bioavailability or toxicity of hazardous components in sediments without sediment removal or engineering attempts. Laboratory study or field scaled evidence must support the natural recovery (NR) potential to restore the site to an appropriate level (Fuchsman et al. 2014; Magar and Wenning 2006). In addition, a modeling effort to ensure that MNR can restore the site in a reasonable time period is essential (Magar et al. 2005; Moore and Langner 2012). Risk management and communication to prevent immediate, unacceptable health risks during the operation of MNR is a crucial step, typically involving fish consumption advisories or water consumption bans. Effective MNR typically requires source zone removal or control (Fuchsman et al. 2014).

In 2013, after 8 years of the official MNR, the Supreme Court ruled in the villagers’ favor (ENLAWTHAI Foundation 2012). According to the verdict, the PCD had been negligent in conducting the remediation plan and mitigation measures as well as an emergency plan after it initially became aware of the pollution in 1998. In addition, the MNR approach would leave people exposed to poisonous Pb in their water for a long time in the future. In this verdict, the court set strict conditions for PCD, that the contaminated environment—water, sediment, aquatic animals, soil, and vegetables in and around the creek—must fall below the acceptable levels on all four occasions over a 1-year period (ENLAWTHAI Foundation 2012). Consequently, PCD started a new round of remedial investigation and feasibility study of Klity Creek restoration again from 2013 to 2015. In the most recent remedial action plan, PCD divided the creek into seven sections (see Figure 1) and will dredge LCS from the creek Sections 2 and 6 only while MNR will be used for the rest of the creek.

For the first time, this study qualitatively and quantitatively evaluated the feasibility of using MNR to clean up the creek. We qualitatively analyzed how LCMT underwent physical and chemical transformations over time in the Klity Creek and evaluated whether such transformations potentially led to NR. Then, we set up a remedial action goal and cleanup levels to protect Klity children from unacceptable Pb poisoning using the integrated exposure uptake biokinetic (IEUBK) model for estimating blood lead levels (BLLs) in Klity children (Hogan et al. 1998) as a function of Pb concentrations in LCS. In addition, we statistically analyzed 10 years of monitoring data of water and sediment samples from eight monitoring stations (Figure 1) and estimated the NR rate constants (*k_NR_*).

## Data Sources, Materials, and Methods

In order to evaluate the NR potential and the appropriateness of using MNR to restore Klity Creek, we first examined the background Pb concentration of Klity sediment using historical records. We then examined the original and transformed characteristics of spilled LCMT over time using the secondary data available from 1998 to 2014. Additionally, we conducted a field sampling of Klity’s water and sediment samples in 2014, followed by physical and chemical characterization to fulfill the comparison of the transformed characteristics over time. Then, we developed the conceptual site model for Pb exposure and formulated appropriate remedial action goals and cleanup levels. Eventually, we quantitatively analyzed the empirical NR rate constants of Pb in water and sediment in the creek over 10 years.

### Sampling and Analysis of Total Pb in Sediment, Water, and Biota

In order to collect the data for the PCD monitoring program over 10 years (from 1998 to 2008) (PCD 2003, 2008, 2009, 2010, 2014), composite bottom sediment samples were collected at a depth of 0–5 cm, using a bottom grab sampler at each sampling station (KC1 to KC8), in a consistent manner. The sediment samples were sieved through a 20-mesh screen (diameter of opening = 0.84 mm). Water samples were collected using a bailer at the middle depth of the creek. The total Pb concentrations in the sediment and water were measured by an accredited PCD laboratory, using atomic absorption spectroscopy (AAS) after sample preparation by acid digestion, according to the U.S. Environmental Protection Agency (EPA) Method 3050B (U.S. EPA 1996) and the U.S. EPA Method 3005A (U.S. EPA 1992), respectively. The sediment samples in 2012 by Poopa et al. (2015) were collected by a bottom grab sampler, digested, and analysed by AAS at Chulalongkorn University, Thailand (Poopa et al. 2015). On the other hand, the bottom sediment samples in 2014 by PCD (2014) were collected by a core tube (7 cm diameter) and sieved through a 20-mesh screen prior to acid digestion and analysed using a graphite furnace AAS by an accredited laboratory at Khon Kaen University, Thailand (PCD 2014). The level of detection (LOD) and level of quantification (LOQ) were 0.876 and 2.920 ppb, respectively, while the percentage recovery was 104.47% (PCD 2014).

Aquatic samples including fish and shrimp [*Metapenaeus affinis* (Jinga shrimp)] were caught at each monitoring station (KC2 to KC8) in 2014, using netting and trapping as well as fishing. The samples were preserved at 0 ± 2°C and digested using nitric acid (HNO_3_) and perchloric acid (HClO_4_) (Walinga et al. 1995). The digested samples were quantified for Pb concentration using a graphite furnace AAS by an accredited laboratory at Khon Kaen University, Thailand (PCD 2014).

### Statistical Analysis of Background Pb Concentration in Klity Sediment and Water

The background Pb concentration in the sediment of Klity Creek that is unaffected by LCMT from the spill or other uncontrolled sources is an important value because, if an NR potential exists, it likely restores the creek back to the background Pb level but not lower. Here, we determined the background value using historical data from multiple sampling locations not affected by LCMT contamination including the upstream (Dee Ka Creek and KC1), the up-elevation (KT4P), and the far downstream (the delta where Ngu Canal intersects Klity Creek) of Klity Creek as summarized in Table S1. Twenty-seven data points from four data sets (PCD 2003, 2010, 2014; Pedall et al. 1999; Poopa et al. 2015) were used to statistically determine the average and the 95% upper confidential level (UCL) of the background Pb concentration. Similarly, the background Pb concentration in Klity’s water was determined from the monitoring data at KC1, upstream of the spill location (KC2) (Figure 1).

### Sieve Analysis and Sequential Extraction of Pb in LCMT and LCS

In order to qualitatively evaluate the transformation of LCMT due to natural processes in the creek, sieve analysis and sequential extraction of Pb were applied to three different types of samples representing three different states of LCMT. Firstly, the LCMT not spilled into Klity Creek was sampled from KT3P and analysed for its particle size distribution, using wet sieve analysis. In addition, the chemical characteristics of Pb in the LCMT were determined, using a three-step sequential extraction procedure developed by the Standards, Measurements, and Testing Programme (formerly the Community Bureau of Reference, BCR). This was done in order to determine four distinct fractions of metals and metalloids in the sediment samples (Brayner et al. 2001; Liu and Shen 2014). Both sieve analysis and sequential extraction were conducted by PCD and reported in 2010 (PCD 2010). The size and chemical fraction of this LCMT sample represented the closest available condition to that of the original LCMT, before spilling into Klity Creek. Secondly, the LCMT sample after 1 year of spill into the creek was obtained from KT2W, which was actually the LCMT dredged from the creek in 1999, by the ore dressing facility. Size analysis and chemical fraction were also obtained from an official PCD report (PCD 2010). The size and chemical fraction of this dredged LCMT sample represent the closest available condition of the LCMT after a 1-year interaction with Klity Creek. Thirdly, the LCS in Klity Creek, 15 years after the spill, was sampled by our research group at KC4 in the zone of still water around 100 m ahead of a check-rock dam in 2013. The core of a sediment sample was collected. Size analysis and chemical fraction were determined in our lab, using the same methods reported in the 2010 PCD report. The size and chemical fraction of this LCS represent the LCMT after 15 years of NR in Klity Creek.

### Remedial Action Goal and Risk-based Cleanup Level Using IEUBK

Prior to evaluating the MNR feasibility, we need to set up an appropriate and achievable remedial action goal and a cleanup level in order to examine whether MNR has the potential to achieve the remedial action goal and cleanup level in a reasonable period of time (Fuchsman et al. 2014). We set up remedial action goals based on regulations, Klity’s geochemical condition, and human health risk associated with Pb in the sediment. In view of the regulations, an appropriate remedial action goal is to clean up the sediment in Klity Creek back to the sediment quality guideline (SQG). Thus, the corresponding cleanup level is 130 mg/kg based on the ecological probable effect concentration of Pb in sediment used in several countries (Burton 2002). In regard to geochemistry, an appropriate remedial action goal is to clean up the sediment in Klity Creek back to the background Pb concentration to be discussed next (see “Background Pb Concentration in Uncontaminated Sediment of Klity Creek” section). On the other hand, in view of human health risk, since the most sensitive targets of Pb poisoning are children in Klity villages, in this study, we set up an appropriate remedial action goal after remediation measures of < 5% of Klity children will be at risk of having a blood lead level (BLL) > 10 μg/dL under their normal lifestyle. This remedial action goal agrees with those goals typically set by the U.S. EPA for other remediations of Pb-contaminated sites (Sheldrake and Stifelman 2003; U.S. EPA 1999, 2011, 2012). In order to derive the cleanup level for this risk-based remedial action goal, we applied the IEUBK model (Hogan et al. 1998) to establish the relationship between Pb concentration remaining in the sediment after remediation and the probable amount of the Klity children’s BLLs.

**Figure 2:**
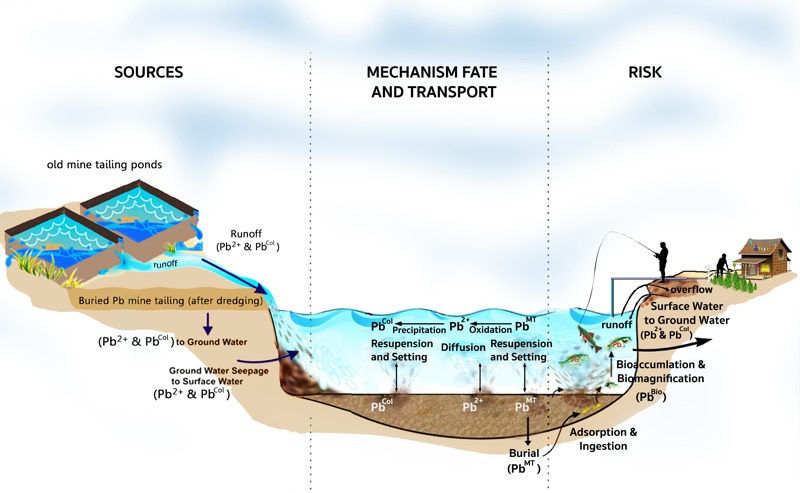
Conceptual site model of Pb contamination in Klity Creek showing Pb sources, five exposure pathways (fish ingestion, drinking water, soil ingestion and dermal contact, edible plant ingestion, and inhalation), fate and transport mechanisms, and human as well as ecological receptors (image courtesy of Raviwan Rakthinkamnerd).

The children of Klity villages may be at risk of Pb poisoning by excess Pb exposure via the following five exposure pathways (Figure 2) considered in our IEUBK model: *a*) directly drinking contaminated water; *b*) ingesting edible plants grown in Pb contaminated soil, or using Pb contaminated water for irrigation; *c*) ingesting fish and other aquatic animals; *d*) being in contact with polluted soil around their houses or playgrounds in the floodplain of the creek; and *e*) breathing air contaminated by Pb-contaminated dust. Nevertheless, the exposure pathways of ingesting contaminated fish and drinking water from the creek are majorly affected by sediment remediation and have been a major concern for the Klity case for more than 17 years. Consequently, we assumed here that the Pb concentrations in air, soil, rice, and edible vegetables grown in Klity remain unaffected by sediment remediation. The most recent Pb concentrations equal 1 μg/m^3^ for the outdoor air (Pusapukdepob et al. 2007), 219 mg/kg in the soil (95% UCL) for Lower Klity village (PCD 2014), 0.32 mg/kg in edible plants (average based on daily consumption of various kinds of vegetables grown in the area) (PCD 2014), and 0.036 mg/kg in rice (PCD 2014), which were used in all the cases of our IEUBK model.

To estimate the probability of the amount of BLLs as a function of Pb in contaminated sediment using IEUBK, we established two relationships: one between Pb in Klity sediment and Pb in drinking water from the creek and one between Pb in Klity sediment and Pb in edible fish in the creek. Since dissolved Pb concentration in Klity Creek is back to the normal background level (10 μg/L) after 20–46 months of the spill (to be discussed in the “Analysis of MNR of Pb in Water” section), Klity children are still exposed to Pb from Pb in contaminated sediment dispersed in drinking water from the creek. We then estimated the Pb concentrations in the drinking water as a function of Pb concentration in the sediment and turbidity of the drinking water. To develop this relationship, LCS from Klity Creek (KC5) was mixed with water from Klity Creek (KC5) to yield the particle concentration of 1–100 mg/L. A turbidity meter was used to measure the turbidity of water samples at different LCS concentrations in water. The correlation between LCS concentrations and turbidity values was developed, and we assumed that if water from Klity Creek is to serve as drinking water, its turbidity should not be greater than 5 nephelometric turbidity units (NTU); otherwise, it will be too objectionable to drink.

Similarly, Pb in fish is theoretically proportional to Pb in the sediment of Klity Creek. This is the concept of the bioconcentration factor (BCF) defined in Equation 1.


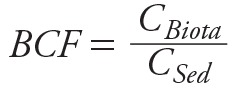
 (1)

Thus, we developed the correlation using the database of *M. affinis* [147 data sets covering all monitoring stations (KC2 to KC8)]. We then derived BCF for fish by scaling the BCF of *M. affinis* with the average Pb concentration in fish and the average Pb concentration in *M. affinis*. This scaling is appropriate under the assumption that in Klity Creek, Pb concentrations in fish and *M. affinis* are proportional to Pb concentrations in the sediment, and there is a constant ratio between Pb concentrations in fish and *M. affinis* at the same location in Klity Creek.

Based on regulatory, geochemical, and risk considerations, an appropriate and achievable remedial action goal was selected. This remedial action goal had to comply with the most important criterion of the nine selected for remedial evaluation: The selection of the criteria was regulated under the Comprehensive Environmental Response, Compensation, and Liability Act 1980 (CERCLA 1980).

### Statistical Evaluation of MNR

In order to evaluate the NR rate of water and sediment, the 10-year data of water and sediment monitoring in Klity Creek were obtained from an official report by PCD (2009). Water and LCS samples from KC1 to KC8 (Figure 1) were collected from 1998 to 2008. In general, the sediment samples were collected in the dry season (mid-February to mid-May), rainy season (mid-May to mid-October), and winter season (mid-October to mid-February). Twenty-one to 23 data points were available for KC1 to KC5, while 11–14 data points were available for KC6 to KC8. The PCD never interpreted the NR potential or NR rate of water or sediment from the monitoring data even 6 years after its publication (PCD 2009). Up to now, there is still no scientific and systematic evaluation of the appropriateness of using MNR for Klity Creek. Here, we evaluated the NR rate constants of water and sediment using the natural decay model (Equations 2 and 3):

For water: C__t__^Pb–W^ = C__B__^Pb–W^ + C__S__^Pb–W^e^–k_NR–W*_t^, (2)

For sediment:C__t__^Pb–Sed^ = C__S__^Pb–Sed^e^–k_NR–Sed*_t^, (3)

where, *C_t_^Pb–Sed^* (mg/kg) and *C_t_^Pb–W^* (mg/L) are the concentrations of Pb in sediment and water, respectively, in each station at a particular time *t* (month), and *C_s_^Pb–Sed^* (mg/kg) and *C_s_^Pb–W^* (mg/L) are the concentrations of Pb in sediment and water, respectively, at the initial time of the spill. The variables *k_NR–Sed_* and *k_NR–W_* are the NR rate constants (month^–1^) of contaminated sediment and water, respectively, while *C__B__^Pb–W^* represents the background Pb concentration in water at each station. This empirical natural decay model was successfully used to model the NR rate of sediment contaminated by metals, including arsenic (As), cadmium (Cd), copper (Cu), Pb, and zinc (Zn) in a recent study (Moore and Langner 2012). Sigmaplot (Systat Software Inc., San Jose, CA) was used for statistical analyses. Standard exponential decay regression was used to determine fitting parameters for each monitoring station using the 10-year data. The *R*, *R* ^2^, and 95% confidence interval (CI) for each fit were determined.

## Results and Discussion

### Background Pb Concentration in Uncontaminated Sediment of Klity Creek

According to the four sampling locations from the five sampling events (see Table S1), 27 data points yield the best-estimated average and 95% UCL background Pb concentrations in the sediment of Klity Creek as 211.99 ± 192.28 and 562.90 mg/kg, respectively. The evaluated natural Pb concentrations in Klity Creek are much higher than the Pb background concentrations in other uncontaminated and non-mining zones in Thailand. For example, Thailand’s Department of Agricultural Extension conducted a survey of 318 soil and sediment samples throughout the country and reported that the mean Pb concentration was 17.50 mg/kg, while the 95% UCL is 54.60 mg/kg, respectively (OAEDG 2014). This comparison confirms that Klity Creek is in the vicinity of a natural Pb occurrence zone and is naturally contaminated up to a level of 200–500 mg/kg of Pb.

It should be noted that we ignored the background Pb concentrations proposed by Pedall et al. (1999) that were taken 9 months after the spill. They distinguished 10 *in situ* uncontaminated Klity sediments from the other 45 illegally discharged LCMT, using the visual difference in physical characteristics and texture between them. According to their survey, the average and 95% UCL background Pb concentrations were 1779.20 ± 1758.70 and 4,627 mg/kg, respectively (see Table S1). These values are much higher than the values determined from the 27 data points discussed earlier. It is highly possible that during the 9 months prior to this survey, LCMT might have infiltrated into, or have been mixed with, the underlying uncontaminated sediment. Since the LCMT was very fine (< 0.1 mm) as a result of milling for ore flotation, it might be difficult to distinguish the fine LCMT mixed into the matrix of the underlying contaminated sediment of Klity Creek. This may explain the elevated Pb concentrations in the sediment samples defined as background Pb concentration by Pedall et al. (1999).

Similarly, Figure S1 illustrates Pb concentrations in water at KC1, around 0.5 km upstream from the point of spill, at KC2 (Figure 1), which can be considered the natural background Pb concentration of the creek. According to the 10-year monitoring data, the total concentration of Pb in water is 0.012 ± 0.017 and 0.039 mg/L for the average and 95% UCL concentrations, respectively. However, the dissolved Pb concentration in water is 0.006 ± 0.002 and 0.01 mg/L for the average and 95% UCL concentrations, respectively. Thus, only 25% of Pb in water is in dissolved form, while the rest is in particulate form, likely to be colloidal hydrocerrusite [Pb_3_(CO_3_)_2_(OH)_2_] formed in water with high carbonate concentration (bicarbonate alkalinity = 143.4 and carbonate alkalinity = 15.6 mg/L as CaCO_3_ in Klity Creek) according to geochemical speciation modeling using MINEQL+ (version 4.5; Environmental Research Software, Hallowell, ME). Notably, the background Pb concentration in Klity Creek’s water is relatively high, but still below the acceptable level of PCD’s surface water quality and drinking water quality standards (0.05 mg/L).

### Evidence of LCMT Transformations in Klity Creek over 16 years

Figure S2 illustrates the most recent Pb concentration in LCS over 28 km of Klity Creek (PCD 2014). Interestingly, even after more than 16 years, Pb concentrations in the sediment are still extremely high [i.e., as high as 160,000 mg/kg close to the spill location (KC2)], much higher than the background Pb concentration discussed previously. Nevertheless, during the 16 years of the interaction between spilled LCMT and Klity Creek, various abiotic and biotic transformations took place. Some processes may attenuate the toxicity of the LCMT, while the others may increase the human health risk as they proceed. Here, we discuss the evidence of LCMT transformations in Klity Creek. To do so, we first present the original characteristics of the LCMT prior to any interaction with the Klity Creek. We then evaluate the characteristics of LCMT after interaction with the Klity Creek at different periods of time to determine the LCMT transformations possibly driven by the creek.

The LCMT was the residual of dressing Pb crude ore from the Bo Ngam mine (around 10 km from the facility) consisting of galena (PbS), cerrusite (PbCO_3_), and some anglesite (PbSO_4_) in limestone, clay, and quartz matrices. In the dressing process, Pb minerals (concentrate), mostly PbCO_3_, were separated from tailings, including clay, quartz, and limestone via the processes of milling (to < 0.1 mm), sulfidization (using Na_2_S), and flotation [using potassium-amyl-xanthate (KAX), dispersant, and pine oil] (Pedall et al. 1999). The size reduction process helps expose Pb minerals in the limestone or quartz matrix to sulfidization for flotation. Sulfidization helps transform PbCO_3_ to PbS, which can subsequently be complexed and hydrophobized by KAX. The KAX hydrophobized ores were then floated by adhering to pine oil, dispersant, or air bubbles. The floated ores were separated from LCMT, which sank to the bottom of the separator and was pumped to the mine tailing ponds. The Pb content in the concentrates was around 65%, while the Pb concentration in the tailing was around 3–4% (i.e., 30,000 to 40,000 mg/kg) by weight on average.

**Figure 3:**
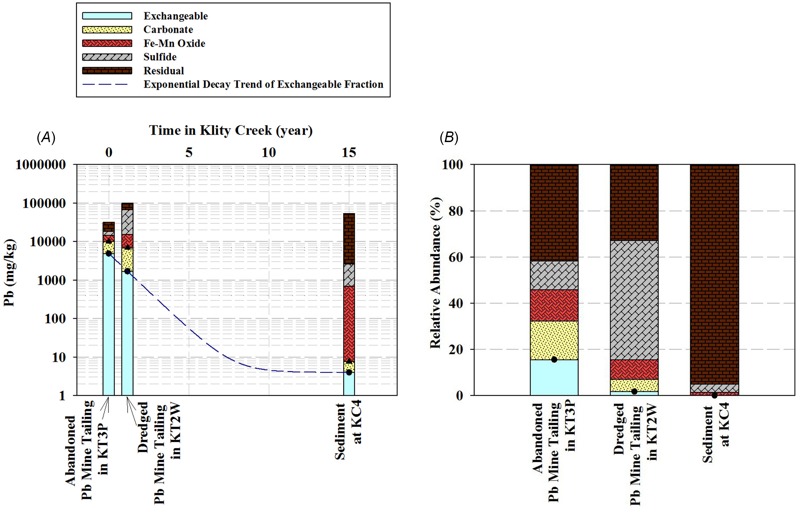
Chemical fractions of Pb in the abandoned mine tailing from KT3P, dredged Pb-contaminated mine tailing from KT2W, LCS in the Klity Creek 15 years after the spill (KC4) reported in (*A*) Pb concentration (mg/kg) and (*B*) percentage of relative abundance.

Figure S3 illustrates the size distribution of LCMT from KT3P. Notably, its size is much smaller than the size of uncontaminated sediment from KT4P (the natural sediment) confirming that the LCMT is small in size as a result of milling during the ore dressing process. Figure 3 illustrates chemical fractions of Pb in the abandoned LCMT from KT3P. Obviously, the LCMT contained a relatively high exchangeable fraction and carbonate fraction, the two most mobile and bioavailable forms (i.e., 4,886 and 5,239 mg/kg, respectively). In addition, the LCMT also has a substantial portion of sulfide fraction (3,908 mg/kg), which can be further weathered (oxidized) in Klity Creek as discussed next. Nevertheless, the LCMT has high concentrations of Pb in residual fraction (13,186 mg/kg). This may be unexposed Pb in the quartz matrix. Several phenomena due to the river mechanism might promote sediment weathering and eventually expose Pb minerals (either PbS or PbCO_3_) inside this residual fraction to the environment.

First, advection of water through the creek might flush LCMT to the Srinagarind Dam (Figure 1) where large amounts of water might be sufficient to dilute Pb contamination to the point that Pb risk to humans becomes acceptable in the dam. As a matter of fact, the operation of advection as an NR mechanism is evident by comparing the change of particle size distribution of LCS at different interaction times with the creek. As shown in Figure S3, the size distribution of LCMT from KT3P (not spilled to the creek) is the smallest (99.9% < 75 μm), while the size of dredged LCMT in KT2W (1-year interaction with the creek) becomes bigger (70% < 75 μm), and the size of LCS at KC4 15 years after the spill is the largest (55% < 75 μm). Presumably, advection of water flushed small mine tailings out of Klity Creek, leaving behind moderate and large mine tailings mixed with natural uncontaminated sediment from upstream (KC1) or with soil from the floodplain. These phenomena can shift the particle size of contaminated sediment to a large size as time proceeds. Interestingly, even 16 years after the spill, elevated LCS is still detected and not fully flushed out of the creek. Presumably, moderate to large LCMT are deposited in zones of still water along the creek. As observed from the nature of the creek, every few dozen meters of the creek are blocked by fallen trees, creating zones of still water upstream of these obstacles. Additionally, Klity Creek has several convex (inner) banks, which favor sediment deposition and accumulation. Moreover, the creek has numerous sinter terraces, which yield zones of still water several hundred meters upstream. Huge amounts of LCS are trapped upstream of the two rock-check dams at KC4 and KC4/1 (Figure 1). The trapped sediment has never been dredged out. Consequently, in this sense, the rock-check dams also serve as long-term sources of LCS, which decrease the efficiency of NR by advection. Further, the 3 mine tailing ponds as well as the 14 improper surface impoundments, burying as much as 900,000 tons of LCMT, may also behave as continuous sources of Pb to the creek over the years after the spill.

In additional to physical processes, abiotic chemical transformation can also promote NR of Pb in sediment. As shown in Figure 3, a substantial fraction of Pb in the original LCMT is exchangeable (i.e., easily dissolved as Pb^2+^). The dissolution of exchangeable Pb decreases Pb concentration in the remaining LCMT, promoting NR of the sediment. Nevertheless, dissolution of Pb from the LCMT to the creek temporarily increases human health risk from this mobile Pb fraction. According to thermodynamic calculation using MINEQL+, cerrusite (PbCO_3_) may be formed after Pb^2+^ is released to a carbonate system of Klity Creek. Similarly, the sulfide fraction of Pb in the spilled LCMT may be oxidatively transformed via interaction with dissolved oxygen in the creek followed by precipitation with carbonate species and eventually forming either colloidal cerrusite particles or cerrusite film covering the PbS surface (Equations 4 and 5) (Lara et al. 2011).

*PbS*(*s*) + 2*O*_2_(*g*) → *PbSO*_4_(*s*) (4)

*PbSO*_4_(*s*) + *H*_2_ *CO*_3_(*aq*) → *PbCO*_3_(*s*) + *H*_2_ *SO*_4_ (5)

After 15 years of interaction with Klity Creek, abiotic transformation of the LCMT is observable. As shown in Figure 3A,B, the LCMT from KT3P (not spilled to the creek) still contains the greatest amount of exchangeable Pb (15.50% or 4,885.70 mg/kg), while the dredged LCMT in KT2W (1-year interaction with the creek) has a smaller amount of exchangeable Pb (1.690% or 1,688.70 mg/kg). The exchangeable fraction of Pb becomes smallest in the LCS at KC4 15 years after the spill (7.56 × 10^–3^% or 3.99 mg/kg). Qualitatively, the exchangeable fraction in the contaminated sediment samples decreases exponentially with respect to the interaction time with the creek. Similarly, the sulfide fraction of Pb in LCMT from KT3P (12.40% or 3,907.8 mg/kg) is much greater than that of the LCS at KC4 (3.59% or 1,895 mg/kg) after 15 years of interaction with the creek, which is in good agreement with the assumption of oxidative transformation by the creek.

Burial and in-place dilution of spilled LCMT by non-contaminated sediment followed by sediment consolidation is a very important physical phenomenon, which can effectively recover the creek (Fuchsman et al. 2014). On the other hand, sediment resuspension by flow turbulence and bioturbation can seriously retard the NR of the creek. Certainly, these two phenomena operate at Klity Creek to some degree. Nevertheless, we cannot separately evaluate their contribution based on the physical and chemical characteristics of the sediment we have. We must evaluate their roles together with the contribution of other prohibiting or promoting phenomena through the analysis of the NR rate constant (*k_NR–Sed_*).

### Appropriate and Achievable Remedial Action Goal and Cleanup Level

Figure S4 illustrates the linear relationship between turbidity of water (in NTU unit) from Klity Creek and the concentration of dispersible Klity sediment in the same water sample. At 5 NTU, the dispersible Klity sediment concentration in the water is 6.16 mg/L. Thus, at the turbidity suitable for drinking, the higher the Pb concentration in the sediment, the greater the Pb concentration in drinking water. As a result, sediment remediation can decrease Pb exposure via drinking water immediately via decreasing the Pb concentration in the dispersible sediment in the water. Similarly, according to Pb concentrations in sediment from KC2 to KC8 (see Figure S2) and Pb concentrations in *M. affinis* caught in KC2 to KC8 (see Figure S5), by neglecting only two data sets from KC4, we found the linear correlation between Pb concentrations in the two environmental media—the sediment and *M*. *affinis—*using both 50% UCL and 95% UCL Pb levels at each station (see Figure S6). The BCF of *M. affinis* is 1.14 × 10^–3^. Then, we derived BCF for fish by scaling the BCF of *M. affinis* with the average Pb concentration in fish (4.46 mg/kg) (PCD 2014) and the average Pb concentration in *M. affinis* (30 mg/kg) (PCD 2014). This yields the BCF for fish = 1.74 × 10^–4^. It should be noted that BCF in fish is relatively low at Klity Creek in comparison to other contaminated sites (Lithner et al. 1995; Nwani et al. 2010), as presumably Pb is in relatively geochemically stable fraction at Klity Creek (Figure 3).

**Figure 4:**
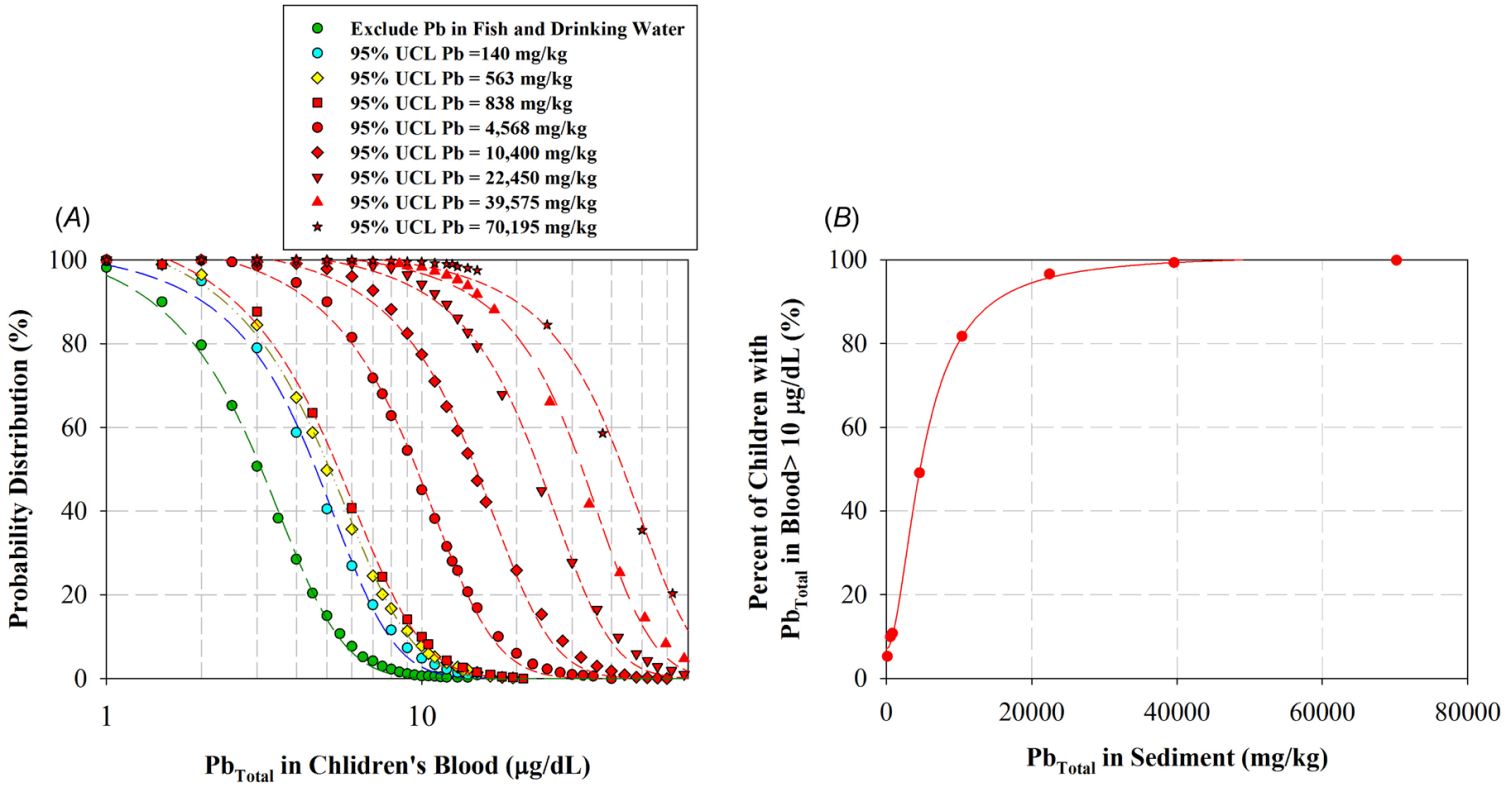
(*A*) The probability of the amount of BLLs of Klity children as a function of 95% UCL Pb in contaminated sediment ranging from 130 to 70,195 mg/kg, and (*B*) the relationship between 95% UCL Pb in contaminated sediment and the percent of Klity children with BLLs > 10.

Now that we have all the correlations ready, we can model the probability of the amount of BLLs as a function of 95% UCL Pb in contaminated sediment ranging from 130 to 70,195 mg/kg (Figure 4A) but first, let us consider the probability distribution of Klity children’s BLLs from the three exposure pathways excluding fish ingestion and drinking water from the creek (dark green circles in Figure 4A). This can be considered the baseline risk from all the exposure pathways not directly affected by sediment remediation. Noticeably, the probability of Klity children having BLLs > 10 μg/dL for this baseline case without the exposure of Pb from fish ingestion and drinking water are only 0.736%, complying with the risk-based remedial action goal. This confirms that Pb exposures via fish ingestion and contaminated drinking water from the creek are two major exposure pathways as indicated by the Ministry of Public Health as well as other stakeholders since 2002.

As theoretically expected, the greater the Pb concentration in the sediment, the greater the probability that Klity children will have high BLLs (Figure 4A). The relationship between 95% UCL Pb in contaminated sediment and the percent of Klity children with BLLs > 10 μg/dL is shown in Figure 4B. In order to achieve the risk-based remedial action goal (i.e., less than 5% of Klity children have BLLs > 10 μg/dL), the Pb concentration in sediment after remediation should be less than 130 mg/kg. For this reason, the cleanup level of 130 mg/kg is suitable, considering both regulatory and risk viewpoints. Nevertheless, it might not be achievable since the background Pb concentration is 563 mg/kg. At this background Pb concentration in the sediment, 7.81% of Klity children will have BLLs > 10 μg/dL, slightly greater than the risk-based remedial action goal. For this reason, the background Pb concentration can be a cleanup level for the revised remedial action goal in that less than 8% of Klity Children will have BLLs > 10 μg/dL after remediation. These remedial action goal and cleanup level are appropriate considering geochemical characteristics of the area, effective enough in terms of risk reduction (7.81% vs. 5%), and more achievable than the 130 mg/kg. In the next section, we will evaluate the feasibility of MNR as a sole remedial approach to restore Klity Creek to achieve the CL of 563 mg/kg.

## Analysis of MNR of Pb in Water

**Figure 5:**
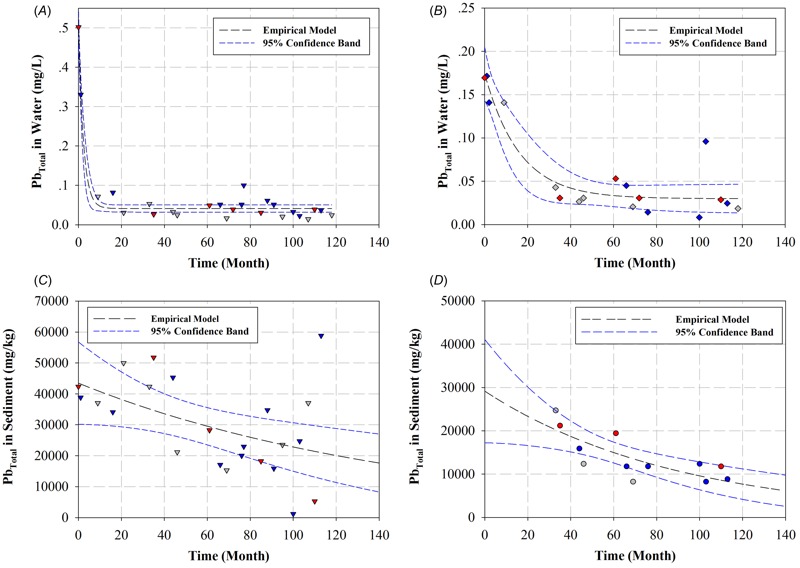
Total Pb concentrations in water at (*A*) KC4 and (*B*) KC7, and total Pb concentrations in sediment at (*C*) KC4 and (*D*) KC8 over 10 years of NR fit by the empirical NR (Equations 2 and 3). Red symbols, blue symbols, and gray symbols represent the sediment samples collected in the dry season (mid-February to mid-May), rainy season (mid-May to mid-October), and winter season (mid-October to mid-February), respectively.

Figures 5A for KC4 and 5B for KC7 (see also Figures S7a for KC2 and S7b for KC5) illustrate the total Pb concentrations in water (both as dissolved and as colloidal) over 10 years. At the beginning of the spill, Pb concentrations were from 0.11 to 0.553 mg/L, while the maximum contamination level of Pb for surface water and drinking water is 0.05 mg/L, according to PCD standards. As a result, the total Pb concentrations in water were 2.2–11.1 times greater than the acceptable level. As shown in Figure 5A,B (see also Figure S7a,b), the 10-year monitoring data of Pb in Klity water, except KC2, are relatively well modelled by the empirical natural recovery model (NRM) in the water phase (Equation 2). The *R* and *R* ^2^ values of the fittings are relatively high, demonstrating a good merit of fit (Table 1). As time proceeded, the Pb concentrations in water were exponentially decreased at all stations (*R* ^2^ from 0.7070 to 0.9656 in Table 1) except KC2 (*R* ^2^ = 0.0832 in Table 1), suggesting the NR of Pb in water. After around 20–33 months, the total Pb concentrations in water were decreasing to a relatively constant value [i.e., 1.92 ± 1.17 × 10^–2^ mg/L (95% UCL = 3.82 × 10^–2^ mg/L) for KC4]. The calculation here excludes the increase of Pb in the water from the 80th to 100th months to be discussed next. These concentrations are similar to the background Pb concentration of Klity Creek (95% UCL = 3.90 × 10^–2^ mg/L in water). Similarly, after around 33–46 months, the total Pb concentrations in water were decreasing to a relatively constant level [i.e., 3.13 ± 1.39 × 10^–2^ mg/L (95% UCL = 5.05 × 10^–2^ mg/L) and 2.87 ± 1.25 × 10^–2^ mg/L (95% UCL = 4.81 × 10^–2^ mg/L)] for KC5 and KC7, respectively. They are in range of the background Pb concentration as well.

**Table 1:** Evaluation of NR and values of fitting parameters based on the empirical MNR (Equation 2) of 10-year monitoring data of Pb in water at KC2, KC4, KC5, and KC7.

Station	NR potential (Y/N)	*R*	*R*^2^	*C*_*B*_^*Pb–W*^ (×10^–^^2^ mg/L)	*C*_*S*_^*Pb–W*^ (×10^–2^ mg/kg)	*k*_*NR–W*_ (×10^–2^ month^–1^)
KC2	N	0.2885	0.0832	4.25 ± 1.27	7.22 ± 5.91	40.64 ± 91.97
KC4	Y	0.9826	0.9656	4.11 ± 0.44	45.69 ± 2.11	43.31 ± 8.12
KC5	Y	0.8408	0.7070	7.61 ± 1.64	49.82 ± 7.58	75.05 ± 26.31
KC7	Y	0.9262	0.8578	2.99 ± 0.79	14.39 ± 1.52	6.17 ± 2.35

The obtained fitting parameters according to Equation 2 summarized in Table 1 make physical sense. It should be noted that the fittings used all the monitoring data, including the fluctuating Pb concentrations during the 80th to 100th months. The values of *C_B_^Pb–W^* (mg/L) are similar to the background Pb concentration, while the values of *C_S_^Pb–W^* (mg/kg) are similar to the initial Pb concentration in water at the beginning of the spill. The values of *k_NR–W_* range from 6.17 ± 2.35 × 10^–2^ to 75.05 ± 2.63 × 10^–2^ month^–1^. Presumably, the NR mechanisms for Pb in water involve *a*) advection of Pb in water to the Srinagarind Dam and *b*) abiotic transformation of Pb in sediment. Since the Pb in water can move with bulk water easily and is much less sensitive to zones of still water than sediment, advection of water through the creek is an effective mechanism to eliminate Pb in water. Similarly, the oxidative dissolution of Pb in sulfide form of the LCMT followed by precipitation as colloidal cerrusite particles or simply the diffusion of exchangeable Pb from the mine tailings to the water column could occur relatively rapidly in the water column or at the sediment–water interface of the creek. Thus, Pb release via abiotic dissolution from the LCMT was expected to be substantial at the early state of the contamination. Over time, the rapidly dissolvable fractions of LCMT were mostly gone (in good agreement with Figure 3), while other moderately dissolvable Pb fractions in mine tailing were buried or diluted in place by natural, uncontaminated sediment. Consequently, the Pb release to water via abiotic dissolution at the water/sediment interface becomes much less severe over time as observed. Nevertheless, during the 80th to 100th months in the rainy season, Pb concentration in sediment increased, presumably due to sediment turn over or resuspension as a result of the greater flow rate in the creek in the rainy season (mid-May to mid-October) (see Figure S8). At the peak of the rainy season, the flow rates can be an order of magnitude higher (Pedall et al. 1999). Similarly, KC2 has the poorest NR potential (unacceptably low *R* and *R* ^2^) with the highest increase of Pb concentration in water again from the 80th to 100th months. Presumably, since KC2 is located closest to the spill, the 3 mine tailing ponds and the 14 improper surface impoundments (Figure 1) may continuously leach Pb out from any of these sources to the creek exceeding the NR potential of the creek. This poor NR potential for KC2 in comparison to other monitoring stations (KC3 to KC8) far from the sources emphasizes the importance of source zone management prior to using MNR.

## Analysis of MNR of Pb in Sediment

Figures 5C (for KC4) and 5D (for KC8) (as well as Figures S9a for KC2 and S9b for KC5) illustrate the total Pb concentrations in sediment over 10 years. At the beginning of the spill, Pb concentrations in the sediment were from 24,705 to 66,470 mg/kg, which are around 43.88–118.06 times greater than the background Pb concentration (95% UCL = 563 mg/kg) or around 190–511 times greater than the SQG (130 mg/kg). Due to plenty of fluctuation in Pb concentrations over time, the Pb concentrations in sediment poorly followed the trend of exponential decrease in all stations except KC8, implying no NR potential of Pb in the sediment. The *R* ^2^ values of the fittings (0.0946–0.2297 in Table 2) for KC2 to KC7 are substantially poorer than the *R* ^2^ values of the previous MNR study for metal contaminated sediment (*R* ^2^ = 0.400, 0.540, and 0.523 for Pb in Deer Lodge, Gold Creek, and Turah Bridge, respectively (Moore and Langner 2012), confirming no significant NR potential for KC2 to KC7. The fluctuation of Pb concentrations in sediment for KC2 to KC7 might be due to the turnover or resuspension of LCS during rainy seasons as observed in the case of water. Nevertheless, it should be noticed that the fluctuation was observed in every season, not only the rainy season. Presumably, the sediment or riverbank sediment, which helps dilute the spilled LCMT, might also be contaminated by Pb leaching from any of the improperly managed sources, making the MNR ineffective. For this reason, KC2, which is closest to the potential Pb sources, has the lowest *R* ^2^. This poor NR potential for KC2 to KC7 again emphasizes the importance of source zone management prior to using MNR.

**Table 2:** Values of fitting parameters based on the empirical MNR (Equation 3) of 10-year monitoring data of Pb in sediment at KC2, KC4, KC5, KC6, KC7, and KC8.

Station	NR potential (Y/N)	*R*	*R*^2^	*C*_*S*_^*Pb–Sed*^ (mg/kg)	*k*_*NR–Sed*_ (×10^–3^ month^–1^)
KC2	N	0.3076	0.0946	53,103 ± 12,253	5.5 ± 3.8
KC4	N	0.4793	0.2297	43,467 ± 6,395	6.5 ± 2.5
KC5	N	0.4544	0.2065	29,283 ± 4,579	5.3 ± 2.4
KC6	N	0.4042	0.1634	24,359 ± 8,019	6.8 ± 4.9
KC7	N	0.4346	0.1889	18,756 ± 4,489	5.9 ± 4.1
KC8	Y	0.7711	0.5945	29,155 ± 5,355	11.1 ± 3.0

Only KC8, farthest from the improperly managed Pb sources and from the point of the spill, has NR potential for the sediment. Presumably, the sediment (riverbank), which helps dilute the spilled LCMT at KC8 might be much less contaminated than other stations, making the NR the most effective. The *R* ^2^ is 0.5945 in line with the *R* ^2^ of the previous study. The obtained fitting parameters for KC8, summarized in Table 2, are reasonable. The value of *C__S__^^Pb–Sed^^* (mg/kg) (29,155 ± 5,355 mg/kg) is similar to the initial Pb concentration in the sediment at the beginning of the spill. The value of *k_NR–Sed_* is 11.1 ± 3.0 × 10^–3^ month^–1^, which is on the same order of magnitude or a little bit higher than the NR rate constants of Pb concentration in Deer Lodge (*k_NR–Sed_* = 2.2 ± 0.5 × 10^–3^ month^–1^), Gold Creek (*k_NR–Sed_* = 3.3 ± 0. 6 × 10^–3^ month^–1^), and Turah Bridge (*k_NR_*_–_ *_Sed_* = 3.4 ± 0.7 × 10^–3^ month^–1^ in the United States, which were reported in a recent study (Moore and Langner 2012). Nevertheless, these rates are around 3.91–6.81 times slower than the NR rate of Klity water for the reasons discussed above (see “Analysis of MNR of Pb in Water” section).

Unlike the water phase, MNR of LCS in Klity Creek was not functioning for KC2 to KC7 and cannot be used as a remediation alternative under the current situation where the potential sources are not properly managed. Nevertheless, MNR appeared to be active at KC8. By transforming the empirical MNR (Equation 3) to Equation 6, we can estimate the time to achieve the cleanup level (*t_CL_*) at KC8, if MNR is used as the sole remedial approach.


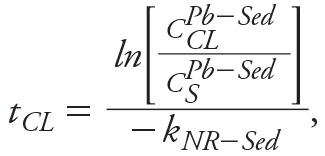
 (6)

where *C__CL__^^Pb–Sed^^* (mg/kg) is the cleanup level in the sediment (563 mg/kg). For KC8, the time to achieve the cleanup level is 377 ± 76 years.

## Conclusion and Future Perspectives

Klity Creek has NR potential for water except at KC2, which is closest to the spill and the other improperly managed Pb sources. Nevertheless, Klity Creek has no substantial NR potential for sediment except the KC8 farthest from the spill and the other sources. For this reason, even the court does not prohibit the use of MNR for Klity cleanup. However, the use of MNR as a sole remedial approach for RDZ1 (KC2), RDZ3, RDZ4, and RDZ5 (KC4 to KC4/1), according to the most recent remediation plan (PCD 2014), will not achieve the court order in that the contaminated environment—water, sediment, aquatic animals, soil, and vegetables in and around the creek—must fall below the acceptable levels on all four occasions over a 1-year period (ENLAWTHAI Foundation 2012). On the other hand, using MNR as a sole remediation approach is theoretically possible to restore RDZ7 (KC8) as planned, but it will require as long as 377 ± 76 years to restore the sediment to the background Pb concentration of the creek. This restoration period is unacceptably long to protect human health from the immediate risk from Pb exposure through fish consumption and drinking water. In addition, dredging only RDZ2 and RDZ6 may not be sustainable since LCS from RDZ1 (KC2) can migrate to re-contaminate the dredged RDZ2, while the LCS from RDZ3, 4, and 5 (KC4 and KC4/1) can migrate to re-contaminate the dredged RDZ6 (KC5 and KC6).

As a result, constructed remediation engineering approaches, such as sediment dredging and sediment capping are essential for restoring the LCS in Klity Creek. The MNR approach will be applicable in some portions of the creek where risk of Pb exposure according to the traditional way of life of the villages is minimal. The way the villagers utilize Klity Creek in daily life must be thoroughly studied in order to evaluate the risk associated with sediment contamination in each portion of the creek according to the different utilization of each portion of the creek. For example, according to an on-going community-based research by Pengkham (2015), Lower Klity villagers catch fish and edible aquatic life from RDZ3, 4, 5, and 7, while they drink water from RDZ5 and RDZ7. These community data must be considered in designing a remedial action plan in order to understand the nature of immediate risk from each section of the creek. Since the current PCD remediation plan does not consider community data, it might use MNR in sections of the creek that can pose immediate risk, such as RDZ3, 4, 5, and 7. More appropriately, MNR can be applied in a train of remediation after a significant portion of highly LCS has been either removed by sediment dredging or capped by sediment capping to the point that no immediate health risks exist. Importantly, to enhance the NR potential of the sediment, the primary sources (3 mine tailing ponds and 14 surface impoundments) as well as the secondary sources, including the Pb-contaminated riverbank should be properly addressed.

## Supplemental Material

(3.8 MB) PDF
