# Supplementary material for: Ten-Year Monitored Natural Recovery of Lead-Contaminated Mine Tailing in Klity Creek, Kanchanaburi Province, Thailand
Source: Environ Health Perspect. 2016 May 8;124(10):1511–20. doi: 10.1289/EHP215 (PMC5047778; doi:10.1289/EHP215)
Supplement: (3.8 MB) PDF [file EHP215.s001.acco.pdf]

Environ Health Perspect
DOI: 10.1289/EHP215

Note to readers with disabilities: *EHP* strives to ensure that all journal content is accessible to all readers. However, some figures and Supplemental Material published in *EHP* articles may not conform to 508 standards due to the complexity of the information being presented. If you need assistance accessing journal content, please contact ehp508@niehs.nih.gov. Our staff will work with you to assess and meet your accessibility needs within 3 working days.

Supplemental Material

Ten-Year Monitored Natural Recovery of Lead-Contaminated Mine Tailing in Klity Creek, Kanchanaburi Province, Thailand

Tanapon Phenrat, Ashijya Otwong, Aphichart Chantharit, and Gregory V. Lowry

Table of Contents

Table S1 Summary of Data used to Determine Background Pb Concentration Klity Sediment

Figure S1. Ten-year monitoring data of total and dissolved lead concentrations in water at KC1 of the Klity Creek. KC1 is around 0.5 km upstream of the point of spill, KC2.

Figure S2. Pb concentration in LCS over 28 km of Klity Creek in 2014.

Figure S3. Size distributions of LCMT (KT3P), dredged LCMT (KT2W), LCS in the Klity Creek 15 years after the spill (KC4), and natural uncontaminated sediment (KT4P)

Figure S4. The linear relationship between turbidity (NTU) of the water from Klity Creek and the concentration of dispersible Klity sediment in the same water sample

Figure S5. Pb concentration in *Metapenaeus affinis* at each sampling station

Figure S6. Linear correlations between Pb concentrations in Klity sediment and Metapenaeus affinis caught at each sampling station using both 50% UCL and 95% UCL Pb levels at each station

Figure S7. Total Pb concentrations in water at KC2 (a) and KC5 (b). Red symbols, blue symbols, and grey symbols represent the sediment samples collected in the dry seasons (mid-

February to mid-May), rainy seasons (mid-May to mid-October), and winter seasons (mid-October to mid-February), respectively.

Figure S8. (a) Average rainfall from 1981 to 2010 in Kanchanaburi's Thong Pha Phum district and (b) maximum rainfall (mm) and day of rainfall (day) from the 36th to 120th month at Kanchanaburi's Thong Pha Phum district.

Figure S9. Total Pb concentrations in sediment at KC2 (a) and KC5 (b). Red symbols, blue symbols, and grey symbols represent the sediment samples collected in the dry seasons (mid-February to mid-May), rainy seasons (mid-May to mid-October), and winter seasons (mid-October to mid-February), respectively.

Table S1 Summary of Data used to Determine Background Pb Concentration Klity Sediment

**Station**	**Year**	**n**	**Pb_{Total} in Sediment (mg/kg)**		**Source**
			**Ave±Std**	**95% UCL**	
Data Included in the Analysis					
KC1	2014	5	328.83 ±275.42	672.8	(PCD 2014)
	2012	2	443.6 ±85.14	497.78	(Poopa et al. 2015)
	2003(upstream)	1	113.50		(PCD 2003)
	2003	3	271.59 ±141.97	410.94	(PCD 2003)
	1998	1	588		(Pedall et al. 1999)
Dee Ka Creek	2014	3	28.83±4.73	33.5	(PCD 2014)
	2003	2	205.20±28.00	223.02	(PCD 2003)
KT4P	2009	7	125.11±116.59	309.91	(PCD 2010)
Ngu Canal	2003	3	101.16±3.83	104.80	(PCD 2003)
Total		27	211.99±192.28	562.90	
Data Excluded from the Analysis					
KC2 to KC8	for 9 months after the spill	11	1779.20±1758.70	4627	(Pedall et al. 1999)

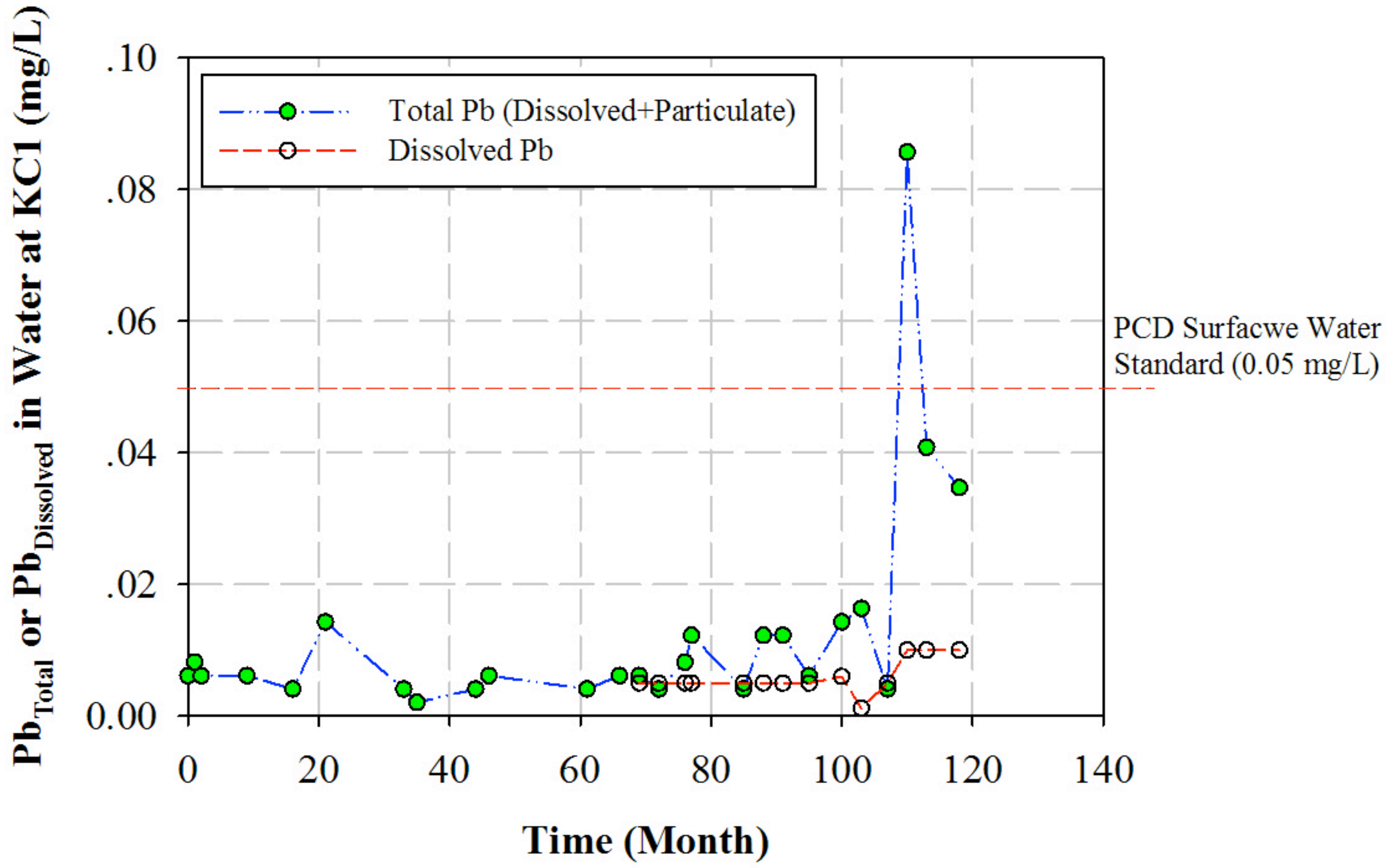

Figure S1. Ten-year monitoring data of total and dissolved lead concentrations in water at KC1 of the Klity Creek. KC1 is around 0.5 km upstream of the point of spill, KC2.

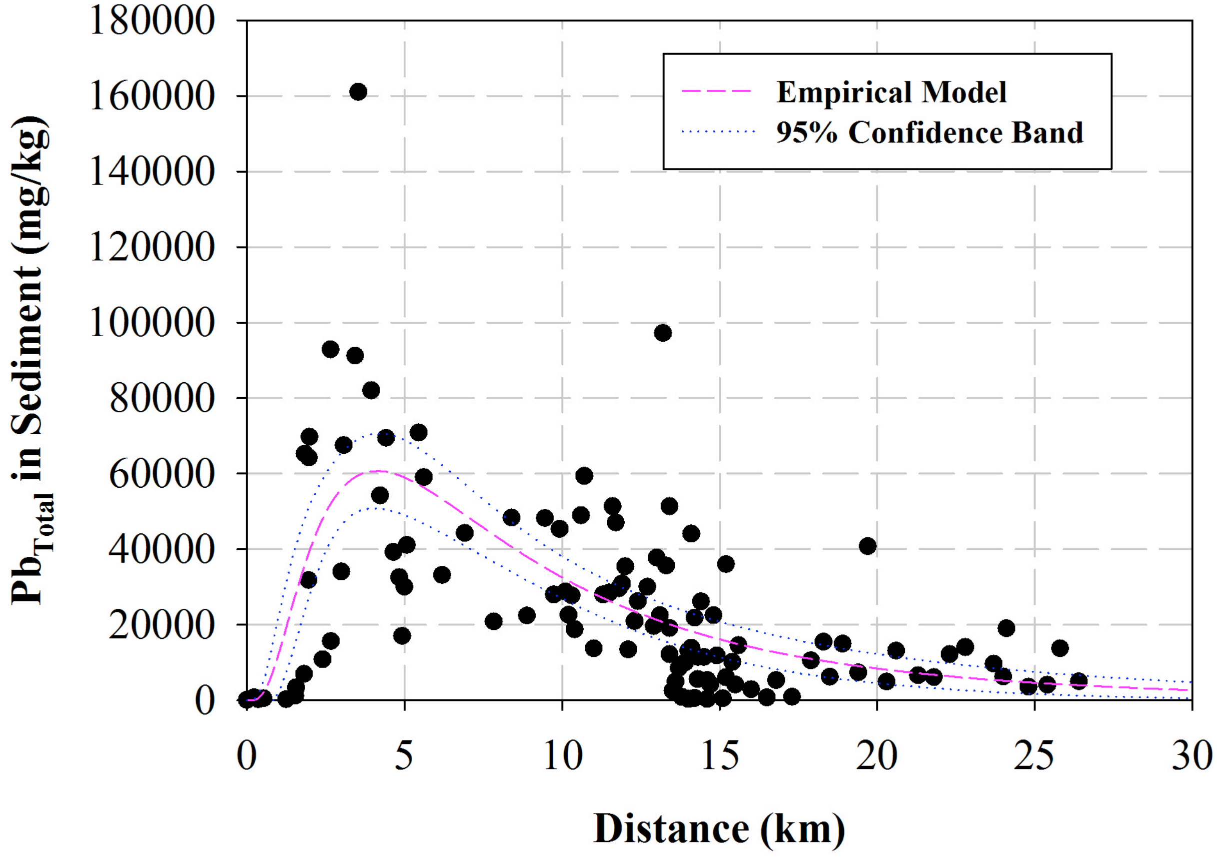

Figure S2. Pb concentration in LCS over 28 km of Klity Creek in 2014.

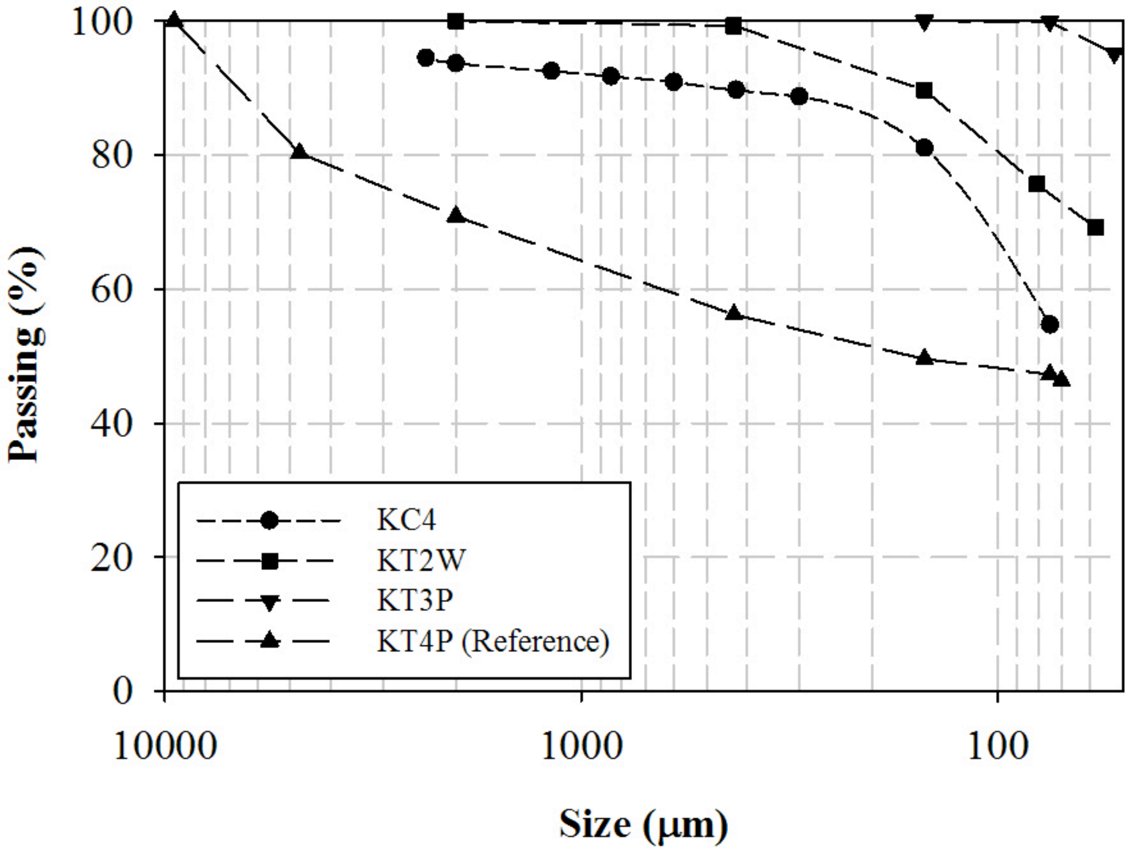

Figure S3. Size distributions of LCMT (KT3P), dredged LCMT (KT2W), LCS in the Klity Creek 15 years after the spill (KC4), and natural uncontaminated sediment (KT4P)

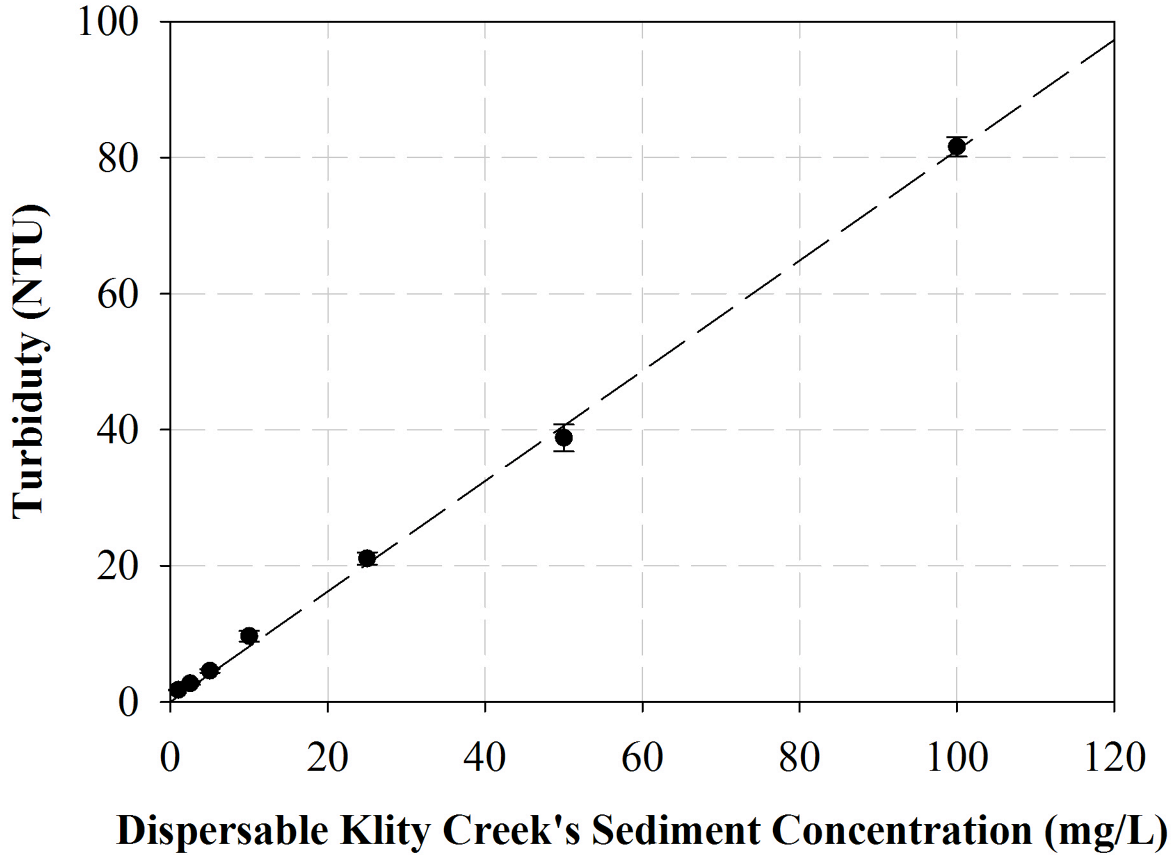

Figure S4. The linear relationship between turbidity (NTU) of the water from Klity Creek and the concentration of dispersible Klity sediment in the same water sample

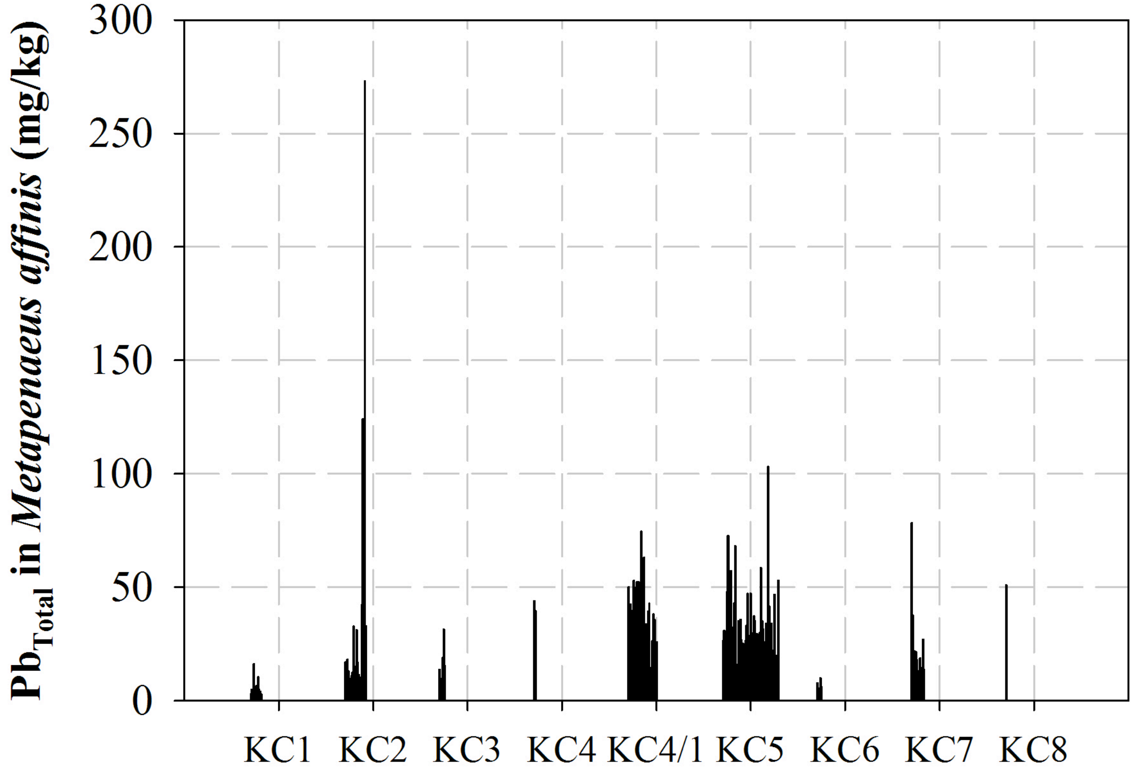

Figure S5. Pb concentration in *Metapenaeus affinis* at each sampling station

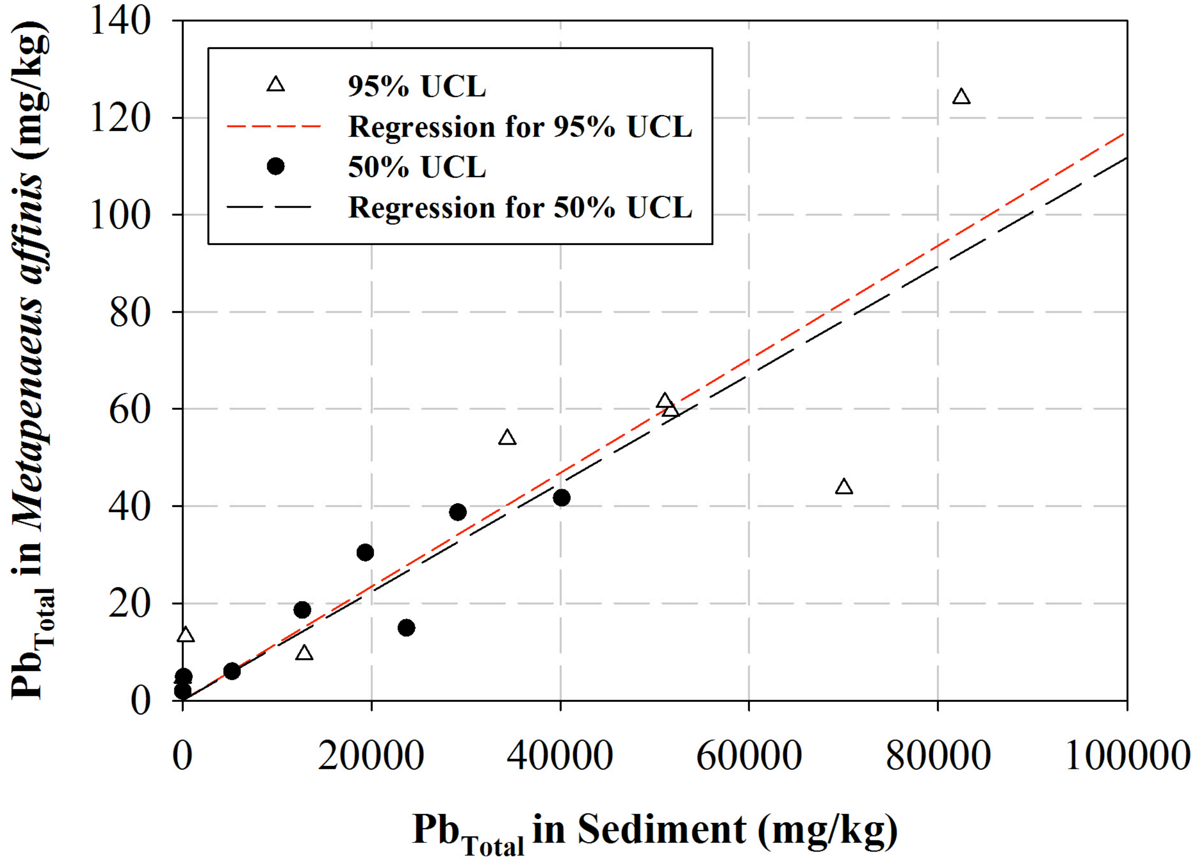

Figure S6. Linear correlations between Pb concentrations in Klity sediment and Metapenaeus affinis caught at each sampling station using both 50% UCL and 95% UCL Pb levels at each station

(a)

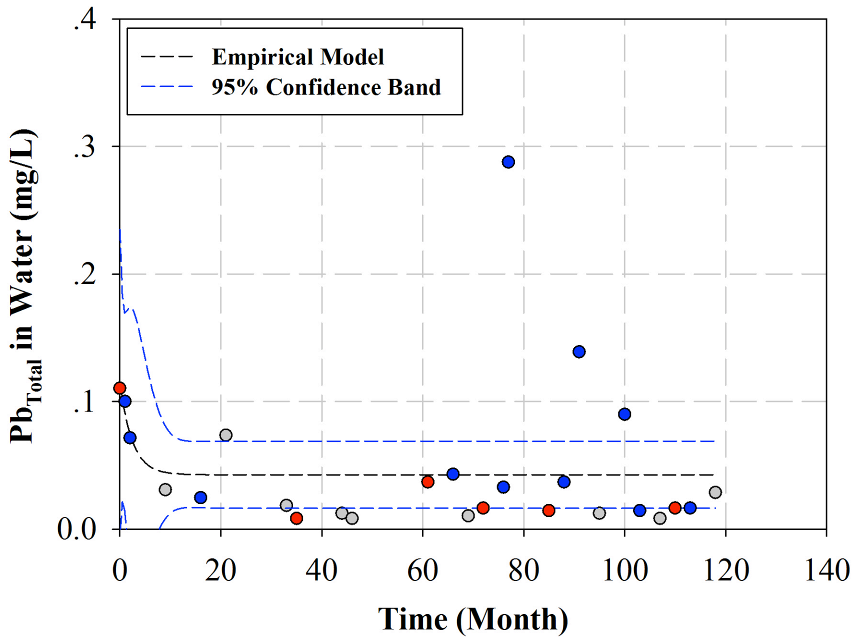

(b)

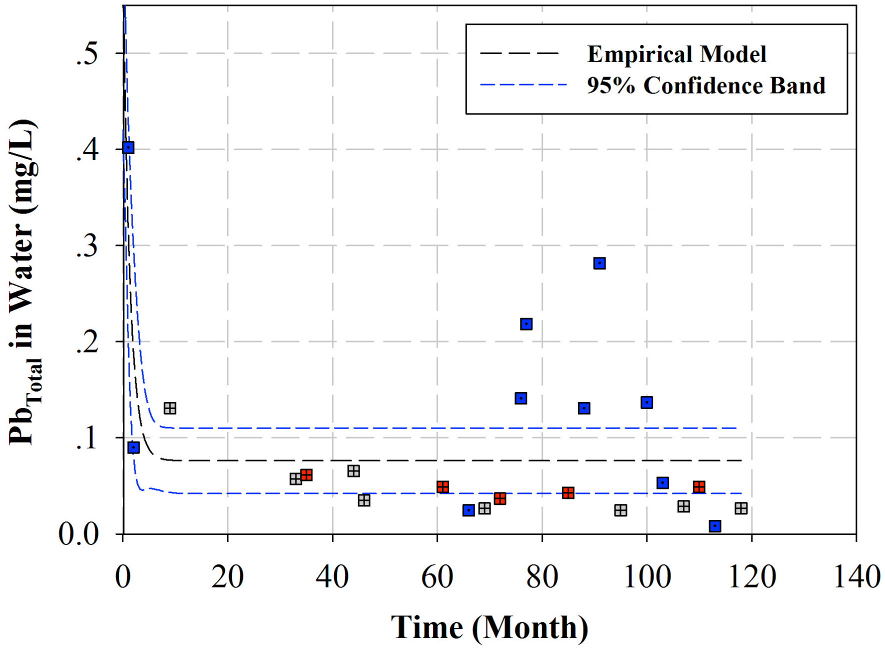

Figure S7. Total Pb concentrations in water at KC2 (a) and KC5 (b). Red symbols, blue symbols, and grey symbols represent the sediment samples collected in the dry seasons (mid-February to mid-May), rainy seasons (mid-May to mid-October), and winter seasons (mid-October to mid-February), respectively.

(a)

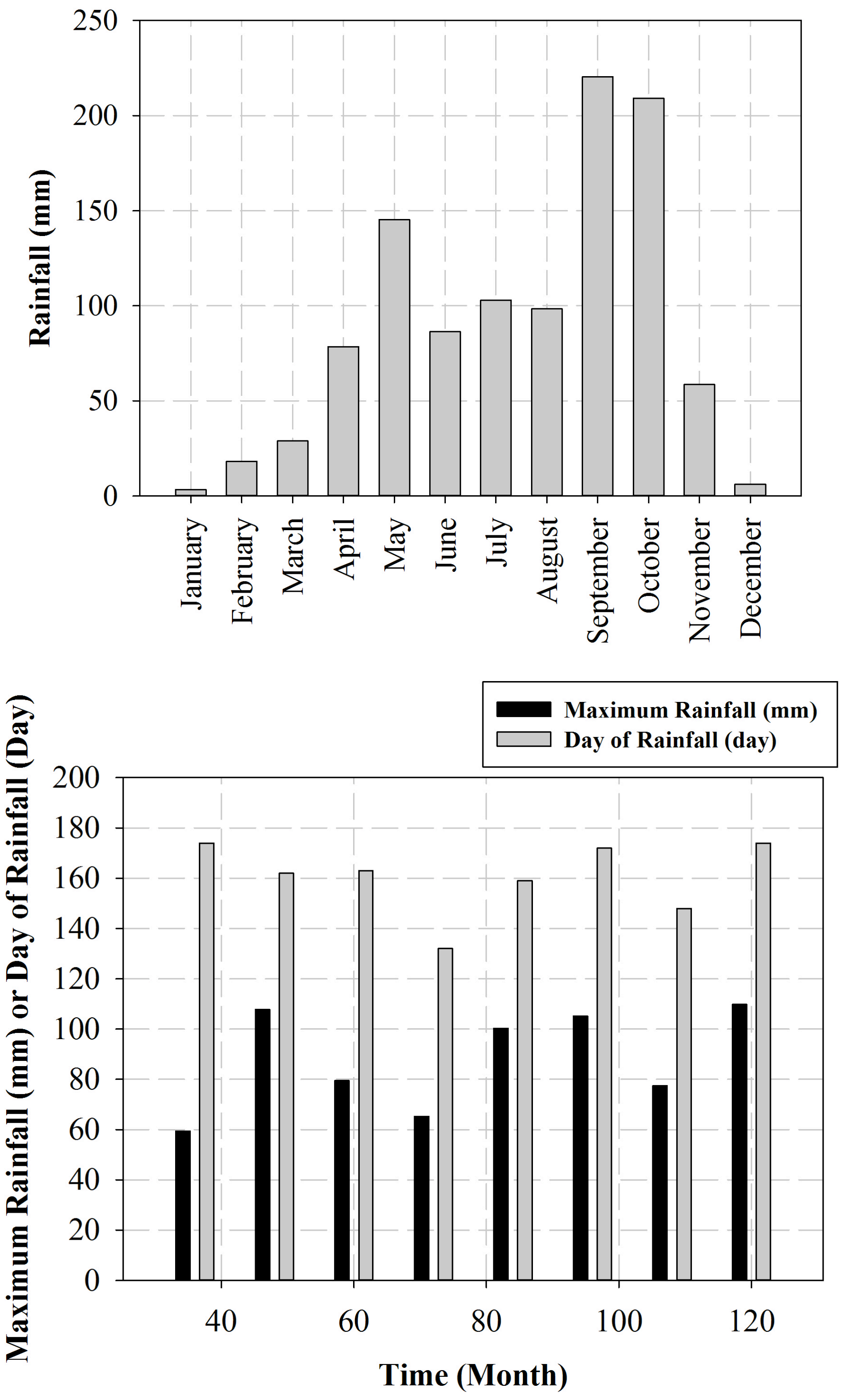

(b)

Figure S8. (a) Average rainfall from 1981 to 2010 in Kanchanaburi's Thong Pha Phum district and (b) maximum rainfall (mm) and day of rainfall (day) from the 36th to 120th month at Kanchanaburi's Thong Pha Phum district.

(a)

(b)

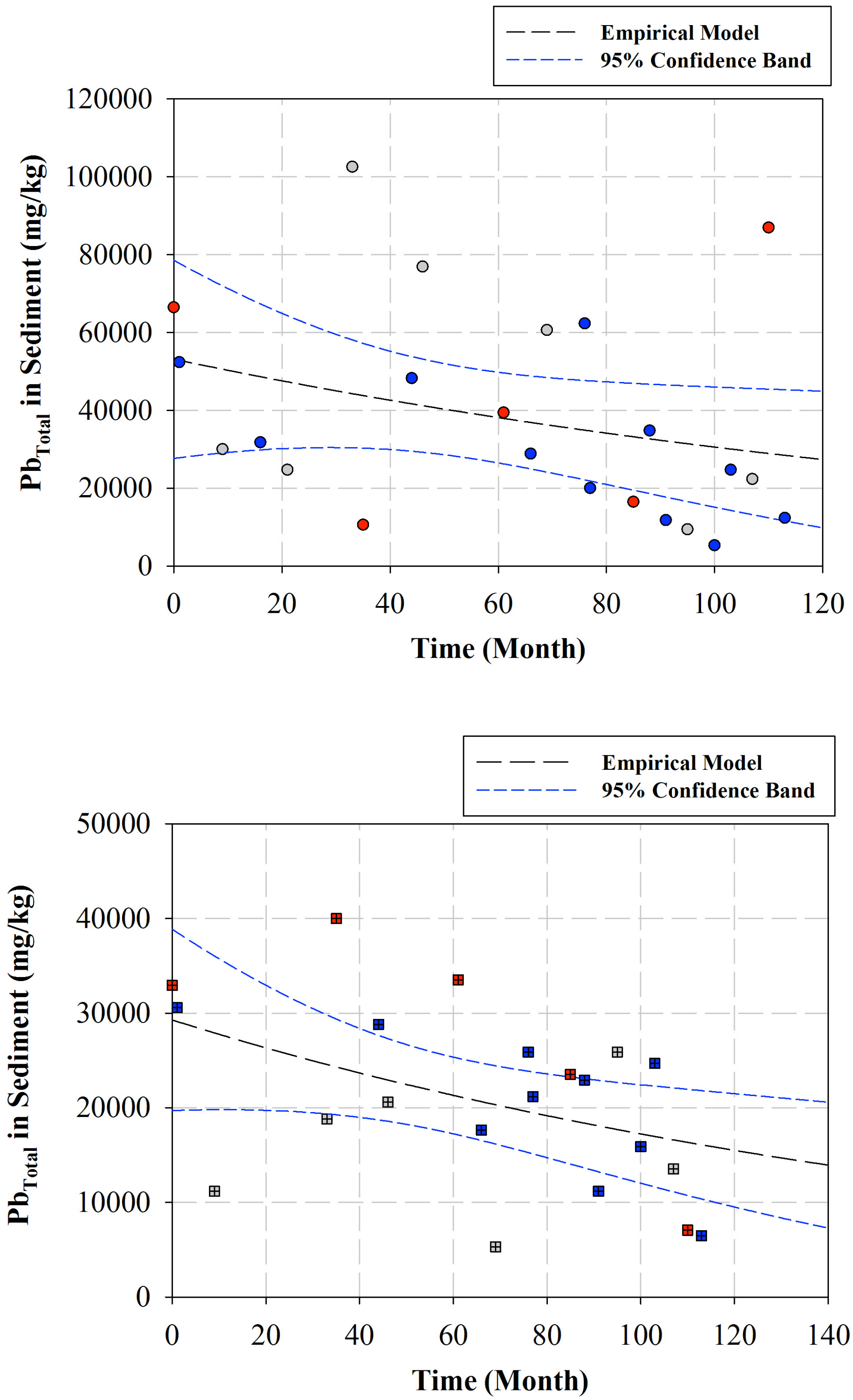

Figure S9. Total Pb concentrations in sediment at KC2 (a) and KC5 (b). Red symbols, blue symbols, and grey symbols represent the sediment samples collected in the dry seasons (mid-February to mid-May), rainy seasons (mid-May to mid-October), and winter seasons (mid-October to mid-February), respectively.

Reference:

PCD. 2003. Site investigation and risk assessment of pb contamination in the upper Mae-Klong watershed. Bangkok, Thailand:PCD.

PCD. 2010. Solving the problem of mine tailing burried along the Klity Creek, Thong Pha Phum district, Kanchanaburi province, Thailand. Bangkok, Thailand: PCD.

PCD. 2014. Feasibility study of cleanup of pb contamination at Klity Creek (Phase I). Bangkok, Thailand:PCD.

Pedall G, Nontaum S, Suthijindawong A. 1999. Lead-contaminated sediments at huay Khi Ti, Thong Pha Phum, Kanchanaburi. Bangkok, Thailand:Lead Concentrates Thailand LCT Ltd.

Poopa T, Pavasant P, Kanokkantapong V, Panyapinyopol B. 2015. Fractionation and mobility of lead in klity creek riverbank sediments, kanchanaburi, thailand. App Envi Res 37:1-10.
